# Idelalisib induces PUMA-dependent apoptosis in colon cancer cells

**DOI:** 10.18632/oncotarget.14043

**Published:** 2016-12-20

**Authors:** Shida Yang, Zhiyong Zhu, Xiaobing Zhang, Ning Zhang, Zhicheng Yao

**Affiliations:** ^1^ Department of Laboratory Medicine, The People's Hospital of Liaoning Province, Shenyang, China; ^2^ Department of Neurology, The People's Hospital of Liaoning Province, Shenyang, China; ^3^ Department of Orthopedics, The People's Hospital of Liaoning Province, Shenyang, China; ^4^ Department of Laboratory Medicine, The First Affiliated Hospital of Liaoning University of Traditional Chinese Medicine, Shenyang, China

**Keywords:** idelalisib, colon cancer, PUMA, apoptosis, GSK3β

## Abstract

Idelalisib, a PI3K inhibitor, specifically targeting p110δ, has been approved for the treatment of chronic lymphocytic leukemia/small lymphocytic lymphoma and follicular lymphoma. However, the mechanisms of action of idelalisib in colon cancer cells are not well understood. We investigated how idelalisib suppresses colon cancer cells growth and potentiates effects of other chemotherapeutic drugs. In this study, we found that idelalisib treatment induces PUMA in colon cancer cells irrespective of p53 status through the p65 pathway following AKT inhibition and glycogen synthase kinase 3β (GSK3β) activation. PUMA is necessary for idelalisib-induced apoptosis in colon cancer cells. Idelalisib also synergized with 5-FU or regorafenib to induce marked apoptosis via PUMA in colon cancer cells. Furthermore, PUMA deficiency suppressed apoptosis and antitumor effect of idelalisib in xenograft model. These results demonstrate a critical role of PUMA in mediating the anticancer effects of idelalisib in colon cancer cells and suggest that PUMA induction can be used as an indicator of idelalisib sensitivity, and also have important implications for it clinical applications.

## INTRODUCTION

Idelalisib, also known as CAL-101 and GS-1101, is the first-in class phosphatidylinositol 3 kinase delta (PI3Kδ) inhibitor, a cytoplasmic tyrosine kinase involved in a number of signaling pathways within B-cells [1]. Idelalisib is approved as a single agent or combined with rituximab (Rituxan) to treat patients with follicular lymphoma, small lymphocytic lymphoma, and relapsed chronic lymphocytic leukemia (CLL) [2–4]. However, idelalisib has a boxed warning regarding serious hepatotoxicity, diarrhea, colitis, intestinal perforation [4, 5]. Idelalisib was found that effectively in CLL patients with p53 mutations who have high risk genetic profiles [4], a finding suggest that idelalisib can be examined at the early time in the course of treatment for patients with p53 deletion/mutations. However, the mechanisms underlying the cell autonomous effect of idelalisib such as cell killing in solid tumors is not well-understood.

PUMA, p53 upregulated modulator of apoptosis, belongs to BH3-only Bcl-2 family, which play a key role in apoptosis in cancer cells [6, 7]. PUMA is a critical mediator of p53-dependent and p53-independent apoptosis in a variety of cancer cell and mice [7, 8]. DNA damage agents such as γ-irradiation, common chemotherapeutic drugs such as 5-fluorouracil (5-FU), induce p53 mediated PUMA induction and apoptosis [9]. The p53-independent manners of PUMA induction by these stimuli is regulated by the transcription factor such as FoxO3a, p73, STAT1, E2F1, or NF-κB, respectively [10–14]. In cancer cells, PUMA induces apoptosis through interact with anti-apoptotic Bcl-2 family members such as Bcl-XL/Bcl-2, which activates the pro-apoptotic members Bax/Bak, resulting in mitochondrial dysfunction and activation of the caspase cascade [6, 15].

Our results demonstrated that PUMA induction by idelalisib via the AKT/GSK-3β/NF-κB pathway and play a pivotal role in therapeutic response to idelalisib in CRC. These results indicated that PUMA induction is indicative of the therapeutic efficacy of idelalisib, and likely other targeted agents as well.

## RESULTS

### Idelalisib induces p53-independent PUMA induction in colon cancer cells

**Figure 1:**
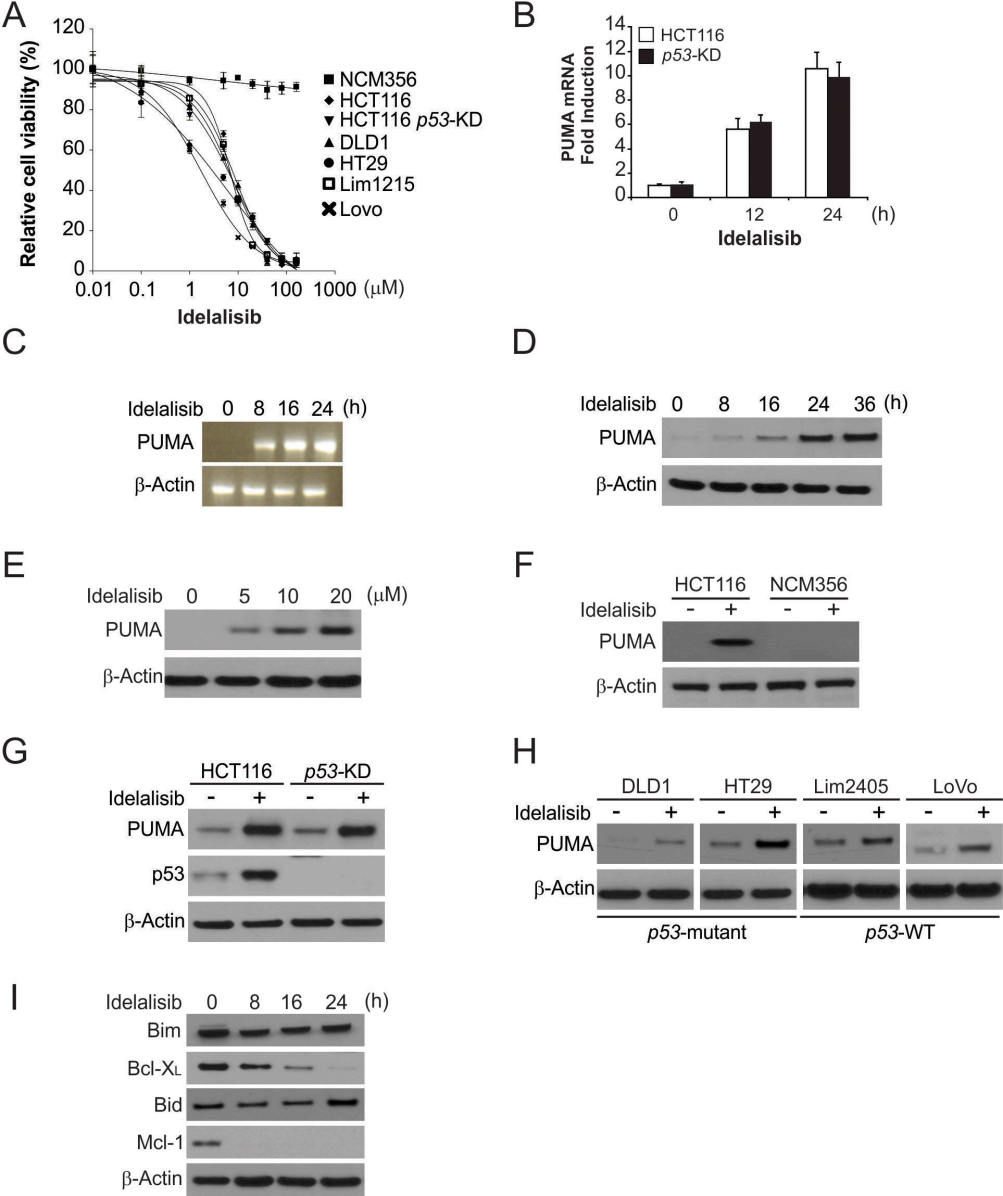
Idelalisib induces p53-independent PUMA induction in colon cancer cells (**A**) Indicated cell lines were treated with different concentrations of idelalisib for 72 hours. Cell proliferation was determined by MTS assay. Results were expressed as means ± SD of three independent experiments. (**B**) Parental and *p53*-KD HCT116 cells were treated with idelalisib at indicated time point. PUMA mRNA induction by idelalisib was analyzed by real-time reverse transcription PCR (RT-PCR), with β-actin as a control. (**C**) HCT116 cells were treated with 10 μmol/L idelalisib at indicated time point. Total RNA was extracted, and PUMA mRNA expression was analyzed by semiquantitive reverse transcription PCR (RT-PCR). β-actin was used as a control. (**D**) HCT116 cells were treated with 10 μmol/L idelalisib at indicated time point. PUMA expression was analyzed by Western blotting. (**E**) HCT116 cells were treated with idelalisib at indicated concentration for 24 hours. PUMA expression was analyzed by Western blotting. (**F**) HCT116 and NCM356 cells were treated with 10 μmol/L idelalisib for 24 hours. PUMA expression was analyzed by Western blotting. (**G**) Parental and *p53*-KD HCT116 cells were treated with 10 μmol/L idelalisib for 24 hours. PUMA expression was analyzed by Western blotting. (**H**) Indicated colon cancer cell lines with different p53 status were treated with 10 μmol/L idelalisib for 24 hours. PUMA expression was analyzed by Western blotting. (**I**) HCT116 cells treated with 10 μmol/L idelalisib at indicated time point. The expression of indicated Bcl-2 family members was analyzed by Western blotting.

To investigate the effects of idelalisib on colon cancer cell lines. We treated 7 colon cancer cell lines with varied concentrations of idelalisib for 72 hours, and then estimated cell proliferation by MTS. We found that idelalisib effectively decreased the cell survival of these cell lines with IC50 ranging from 2 μmol/L to 10 μmol/L (Figure 1A). Treating HCT116 colon cancer cells with idelalisib markedly induced protein and mRNA levels of PUMA in a time- and dose-dependent manner (Figure 1B–1E). Then, we examined the action of idelalisib on NCM356 normal intestinal epithelial cells (IECs), and found that idelalisib did not decrease the proliferation of NCM356 cells and no PUMA induction in the cells (Figure 1A and 1F). Cytotoxic effect of idelalisib in parental and stable p53-Knockdown (*p53*-KD) HCT116 cells is similar (Figure 1A). Idelalisib also induced PUMA protein and mRNA expression in *p53*-KD HCT116 cells (Figure 1B and 1G). Idelalisib induced PUMA expression in other colon cancer cells including Lim2405, LoVo, HT29 and DLD1 cell lines regardless of the p53 status (Figure 1H). In contrast, idelalisib treatment did not upregulate Bid and Bim protein level, but reduced the protein level of the anti-apoptotic such as Bcl-XL and Mcl-1 (Figure 1I). The above data suggested that idelalisib-induced PUMA expression in a p53-independent manner, and PUMA may contribute to the antitumor effects of idelalisib.

### PUMA play a role in idelalisib-induced apoptosis

**Figure 2:**
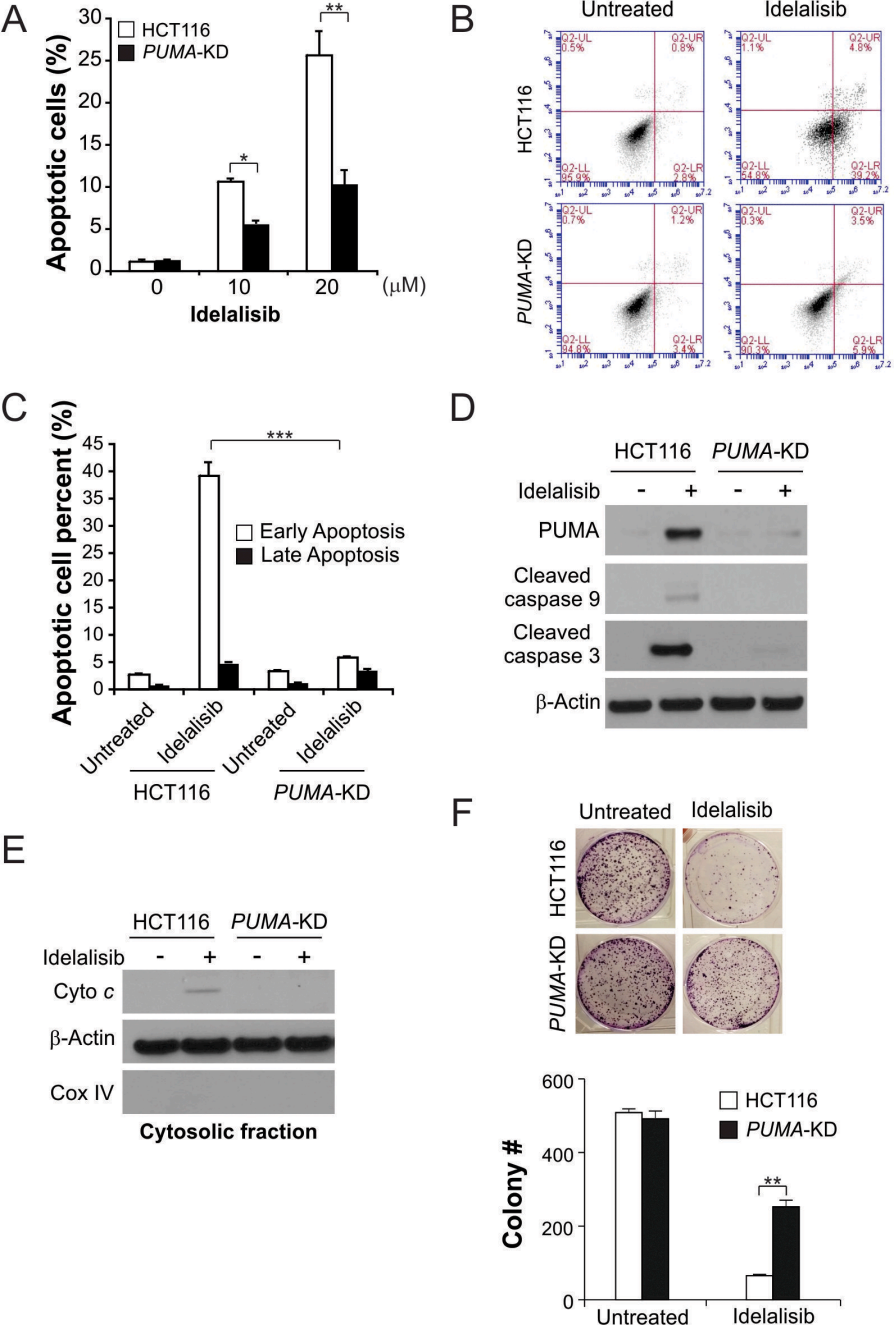
PUMA mediates the anticancer effects of idelalisib through the mitochondrial pathway (**A**) Parental and *PUMA-*KD HCT116 cells were treated with idelalisib at indicated concentration for 24 hours. Apoptosis was analyzed by a nuclear fragmentation assay. (**B**) Parental and *PUMA-*KD HCT116 cells were treated with 10 μmol/L idelalisib for 24 hours. Apoptosis was analyzed by annexin V/PI staining followed by flow cytometry. (**C**) Early apoptotic and late apoptotic cells were analyzed by flow cytometry as treated in (B). (**D**) Parental and *PUMA-*KD HCT116 cells were treated with 10 μmol/L idelalisib for 24 hours. Cleaved caspase 3 and 9 expression were analyzed by western blotting. (**E**) The cytoplasm and mitochondria were fractionated from HCT116 cells that stably expressed scramble shRNA or PUMA shRNA treated with 10 μmol/L idelalisib for 24 hours. The distribution of cytochrome c was analyzed by Western blotting. β-actin and cytochrome oxidase subunit IV (Cox IV) were analyzed as the control for loading and fractionation. (**F**) Parental and *PUMA-*KD HCT116 cells were treated with 10 μmol/L idelalisib for 24 hours. Colony formation assay was done by seeding an equal number of treated cells in 12-well plates, and then staining attached cells with crystal violet 14 days later. Left, representative pictures of colonies; Right, quantification of colony numbers. Results in (A), (C) and (F) were expressed as means ± SD of 3 independent experiments. ***P < 0.001;**P < 0.01; *P < 0.05.

Next, we investigated the potential functions of PUMA in idelalisib-induced apoptosis using PUMA stable knockdown (*PUMA-*KD) HCT116 cells. Idelalisib treatment induced significantly apoptosis in HCT116 cells, which was significantly reduced in *PUMA-*KD cells (Figure 2A). The low PUMA expression in HCT116 cells abrogated idelalisib-induced apoptosis was confirmed by Annexin V/PI staining (Figure 2B and 2C). Idelalisib treatment induced caspase 3 and 9 activation, and cytochrome c release, which was suppressed in *PUMA-*KD cells (Figure 2D and 2E). Furthermore, *PUMA-*KD cells had improved survival than parental HCT116 cells in a long-term cologenic assay following idelalisib treatment (Figure 2F). Therefore, PUMA is necessary for the apoptotic effect of idelalisib in colon cancer cells.

### NF-κB mediated idelalisib-induced PUMA expression

**Figure 3:**
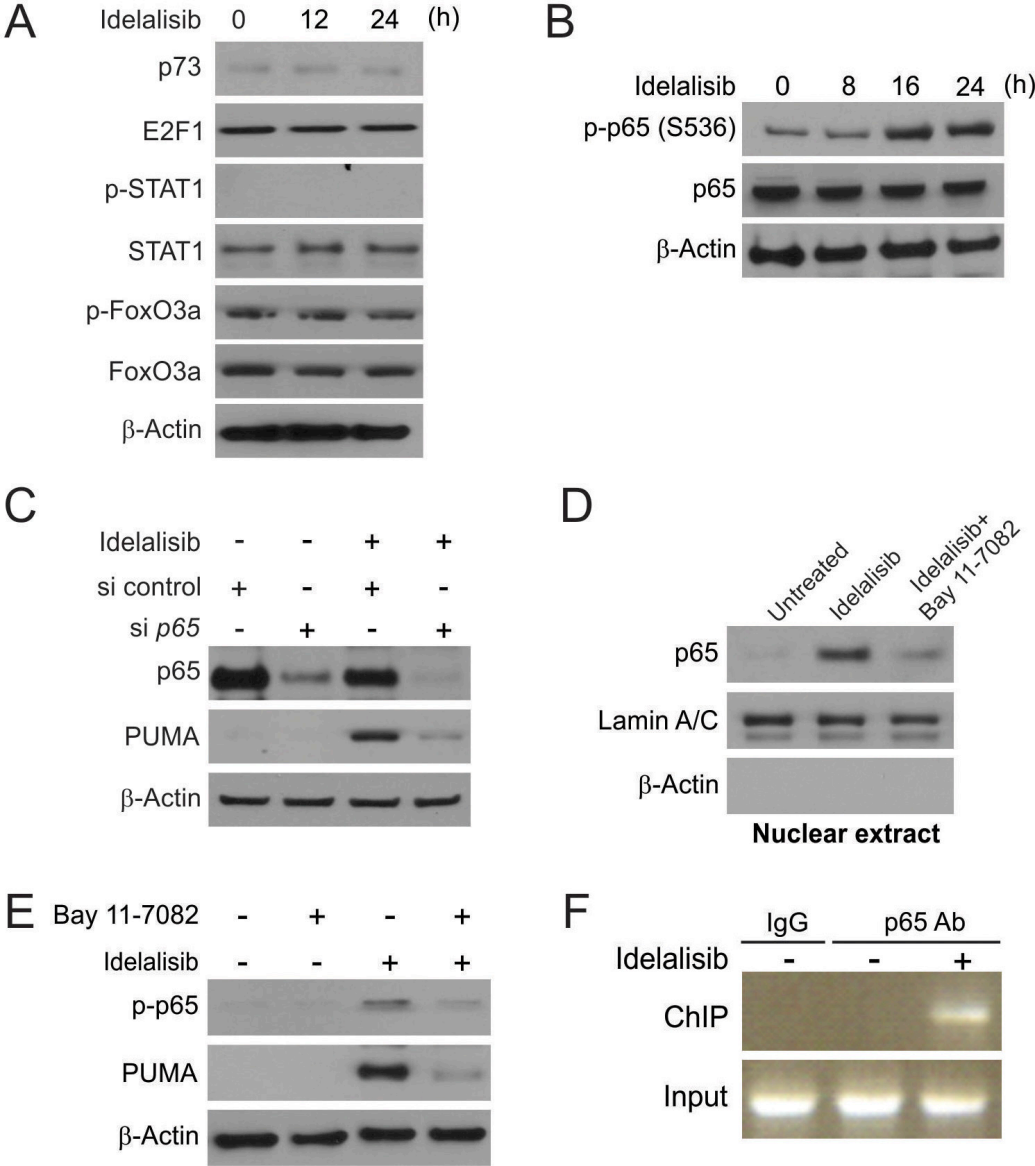
p65 mediates idelalisib induced PUMA induction (**A**) *p53*-KD HCT116 cells were treated with 10 μmol/L idelalisib at indicated time point. p73, E2F1, p-STAT1, STAT1, p-FoxO3a and FoxO3a expression was analyzed by Western blotting. (**B**) HCT116 cells were treated with 10 μmol/L idelalisib at indicated time point. p-p65 (S536) and p65 expression was analyzed by Western blotting. (**C**) HCT116 cells were transfected with either a control scrambled siRNA or a p65 siRNA for 24 hours, and then treated with 10 μmol/L idelalisib for 24 hours. p65 and PUMA expression was analyzed by Western blotting. (**D**) HCT116 cells were pretreated with 10 μmol/L BAY11-7082 for 1 hour, and then with 10 μmol/L idelalisib for 24 hours. Nuclear fractions were isolated from cells and analyzed for p65 expression by Western blotting. Lamin A/C and β-actin were used as controls for loading and fractionation. (**E**) HCT116 cells were pretreated with 10 μmol/L BAY11-7082 for 1 hour, and then with 10 μmol/L idelalisib for 24 hours. p-p65 (S536) and PUMA expression was analyzed by Western blotting. (**F**) Chromatin immunoprecipitation (ChIP) was performed using anti-p65 antibody on HCT116 cells following idelalisib treatment for 12 hours. ChIP with the control IgG was used as a control. PCR was carried out using primers surrounding the p65 binding sites in the PUMA promoter.

We next determined the mechanism of PUMA induction by idelalisib. Several transcription factors, which can mediate PUMA induction in *p53*-KD HCT116 cells, were examined to further delineate the mechanism of PUMA induction. FoxO3a is not involved due to unchanged inhibitory phosphorylation following idelalisib treatment (Figure 3A). p73 and STAT1 were also ruled out due to lack of induction or a change in phosphorylation/activation (Figure 3A).

In previously study, NF-κB was found to transcript expression of PUMA following TNF-α, Aurora Kinase inhibitors or regorafenib treatment [11, 16, 17]. HCT116 cells treated with idelalisib induced phosphorylation of p65 (S536) in a time-dependent manner (Figure 3B). Knockdown of *p65* by transient expression of siRNA suppressed PUMA induction by idelalisib treatment (Figure 3C). Following idelalisib treatment, *p65*translocated to the nucleus, which can be suppressed by NF-κB specific inhibitor BAY 11-7082 (Figure 3D). As shown in Figure 3E, NF-κB inhibition also abrogated PUMA induction and p65 phosphorylation induced by idelalisib, suggesting that p65 activation/nuclear translocation mediated PUMA induction by idelalisib. Next, we investigated whether NF-κB can directly binding to PUMA promoter. The recruitment of p65 to the promoter of PUMA was found following idelalisib treatment by chromatin immunoprecipitation (ChIP) (Figure 3F). The above data demonstrated that p65 regulated PUMA expression by directly binding to multiple κB sites of PUMA promoter following idelalisib treatment.

### GSK3β activation mediated p53-independent PUMA induction following idelalisib treatment

**Figure 4:**
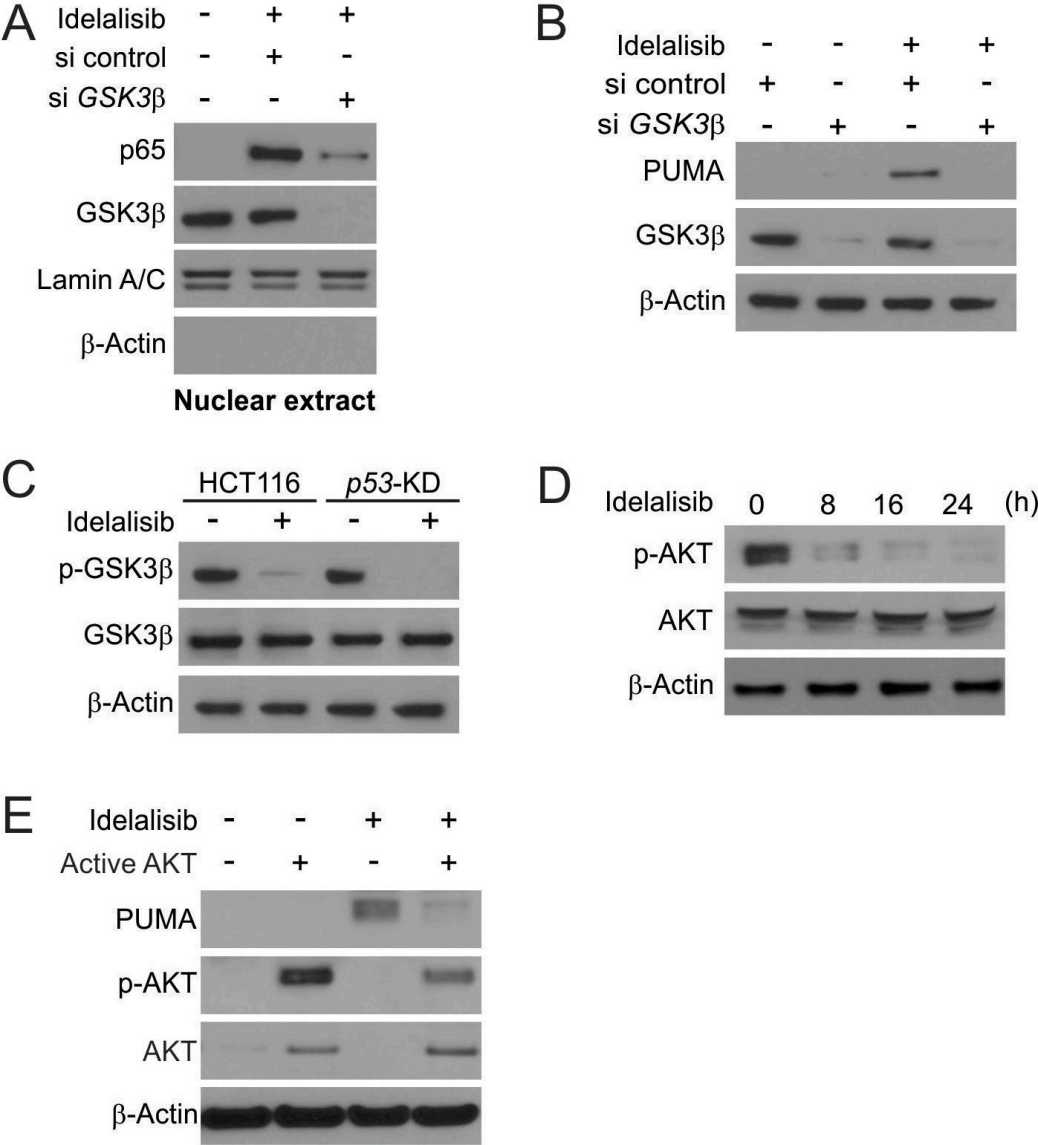
PUMA induction by idelalisib is mediated through GSK3β activation (**A**) HCT116 cells were transfected with either a control scrambled siRNA or a GSK3β siRNA for 24 hours, and then treated with 10 μmol/L idelalisib for 6 hours. Nuclear fractions were isolated from cells treated with idelalisib and analyzed for p65 and GSK3β expression by Western blotting. (**B**) HCT116 cells were transfected with either a control scrambled siRNA or a GSK3β siRNA for 24 hours, and then treated with 10 μmol/L idelalisib for 24 hours. GSK3β and PUMA expression was analyzed by Western blotting. (**C**) Parental and *p53*-KD HCT116 cells were treated with 10 μmol/L idelalisib for 24 hours. Total GSK3β and p-GSK3β (S9) expression was analyzed by western blotting. (**D**) HCT116 cells were treated with 10 μmol/L idelalisib at indicated time point. Total AKT and p-AKT expression was analyzed Western blotting. (**E**) HCT116 cells were transfected with Active AKT plasmid for 8 hours, and then treated with 10 μmol/L idelalisib for 24 hours. PUMA, p-AKT, and total AKT expression was analyzed by Western blotting.

Next, we determined if GSK3β is involved in idelalisib-induced p65 activation. First, we found that GSK3β siRNA but not the control siRNA suppressed idelalisib-induced nuclear translocation of p65 (Figure 4A). GSK3β depletion abrogated idelalisib-induced PUMA induction in HCT116 cells (Figure 4B). Furthermore, parental and *p53*-KD HCT116 were treated with idelalisib. Idelalisib treatment dephosphorylated GSK3β (Ser9), which leading to GSK3β inactivation in both cell lines [18] (Figure 4C). In previously study, AKT can phosphorylate GSK3β on Ser9 site to inhibit its activity [19, 20]. Idelalisib treatment significantly suppressed activation of AKT (Ser473) in time-dependent manner (Figure 4D). Overexpression of constitutively active AKT suppressed idelalisib-induced PUMA induction and p65 activation (Figure 4E). The above data suggested that AKT inhibition mediates GSK3β activation, leading to p65 translocation and PUMA induction by idelalisib.

### PUMA mediates the chemosensitization effects of idelalisib

**Figure 5:**
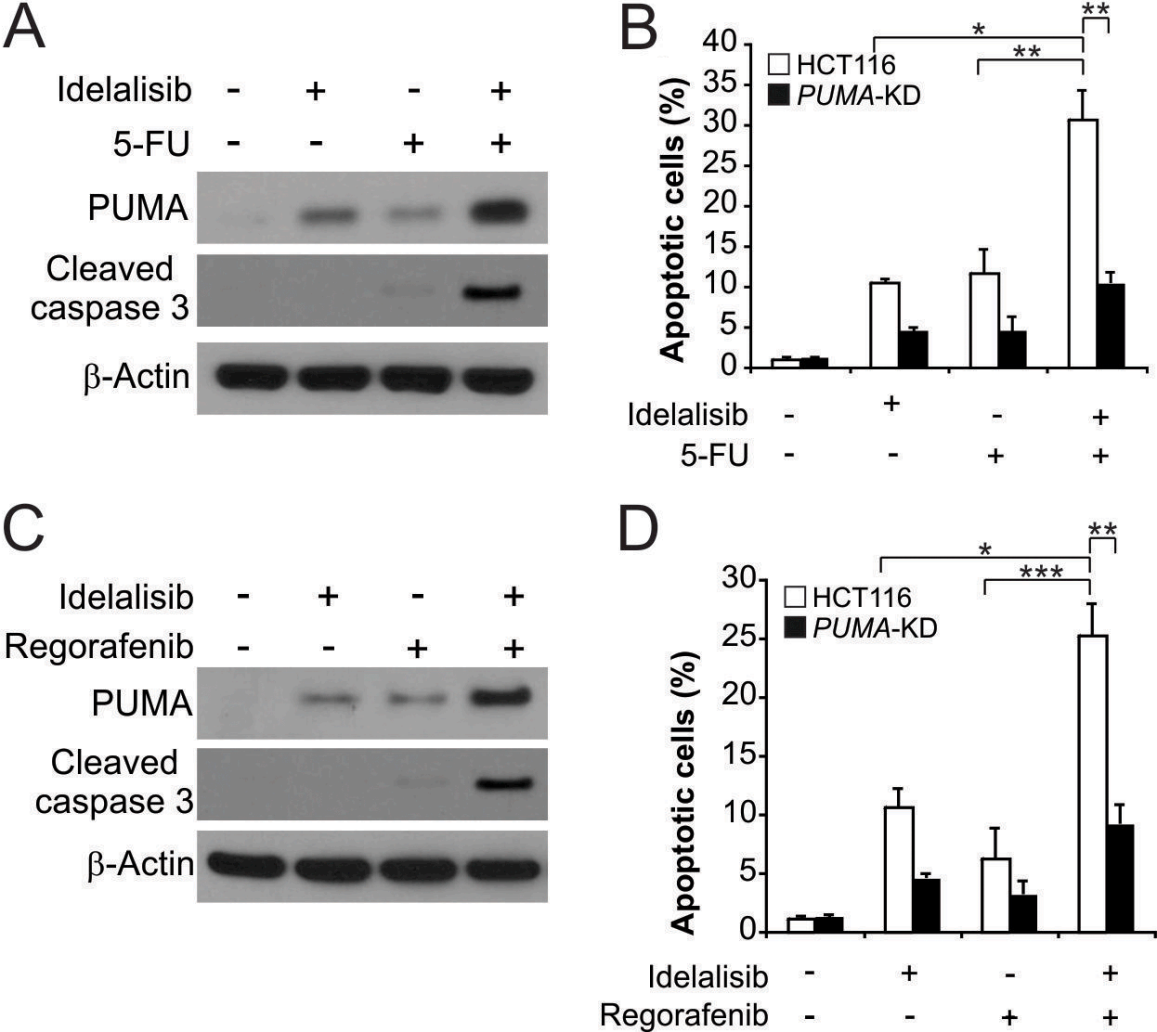
Idelalisib synergizes with 5-FU or regorafenib to induce apoptosis via PUMA in colon cancer cells (**A**) HCT116 cells were treated with 5 μmol/L idelalisib, 20 mg/L 5-FU, or their combination for 24 hours. PUMA and cleaved-caspase 3 expression were analyzed by Western blotting. (**B**) Parental and *PUMA-*KD HCT116 cells were treated 5 μmol/L idelalisib , 20 mg/L 5-FU, or their combination for 24 hours. Apoptosis was analyzed by a nuclear fragmentation assay. (**C**) HCT116 cells were treated with 5 μmol/L idelalisib, 20 mg/L regorafenib, or their combination for 24 hours. PUMA and cleaved-caspase 3 expression were analyzed by Western blotting. (**D**) Parental and *PUMA-*KD HCT116 cells were treated 5 μmol/L idelalisib , 20 mg/L regorafenib, or their combination for 24 hours. Apoptosis was analyzed by a nuclear fragmentation assay. Results in (B) and (D) were expressed as means ± SD of 3 independent experiments. ***P < 0.001;**P < 0.01; *P < 0.05.

The chemosensitization effect of idelalisib has been used in clinical studies [21–23]. The combination of idelalisib with 5-FU induced higher levels of PUMA, compared to single agent alone treatment (Figure 5A). The combination treatment induced higher level of apoptosis and caspase 3 activation in HCT116 cells. However, the combination induced apoptosis and caspase 3 activation were abolished in *PUMA-*KD HCT116 cells. (Figure 5A and 5B). Furthermore, the PUMA-dependent sensitization effect was also observed in cells treated with idelalisib combined with the regorafenib (Figure 5C). The combination treatment induced higher level of apoptosis and caspase 3 activation in HCT116 cells. However, the combination induced apoptosis and caspase 3 activation were abolished in *PUMA-*KD HCT116 cells. (Figure 5C and 5D). These findings demonstrated a general role of PUMA in the chemosensitization effects of idelalisib in colon cancer cells.

### PUMA contributes to the antitumor activity of idelalisib in a mouse xenograft model

**Figure 6:**
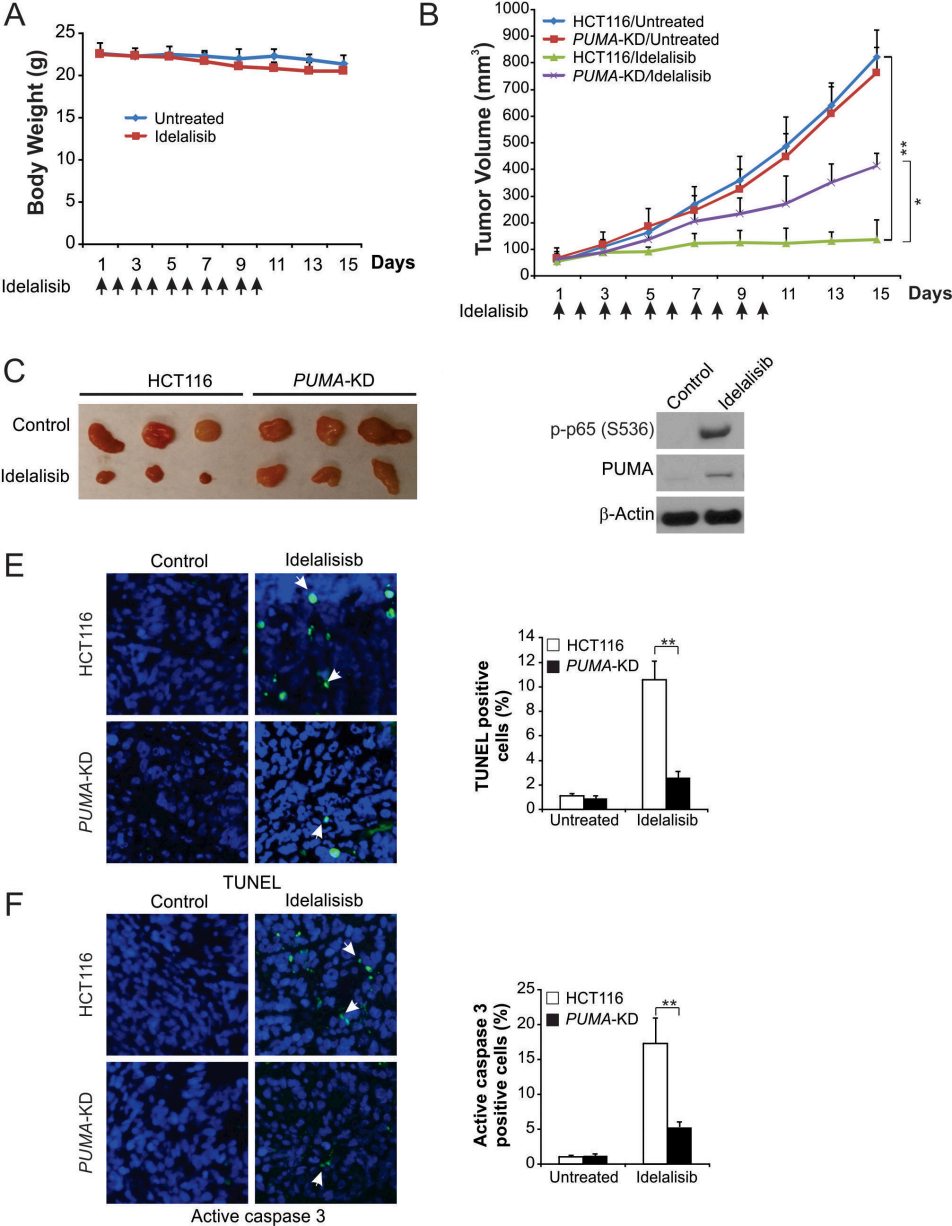
PUMA mediates the antitumor effects of idelalisib in a xenograft model (**A**) Nude mice were treated with 30 mg/kg idelalisib for 10 consecutive days. Body weight at indicated time points was measured. Arrows indicate idelalisib injection. (**B**) Nude mice were injected s.c. with 4 × 10^6^ parental and *PUMA-*KD HCT116 cells. After 1 week, mice were treated with 30 mg/kg idelalisib or buffer for 10 consecutive days. Tumor volume at indicated time points after treatment was calculated and plotted (*n* = 6 in each group). Arrows indicate idelalisib injection. (**C**) Representative tumors at the end of the experiment in (B). (**D**) Parental HCT116 xenograft tumors were treated with 30 mg/kg idelalisib or the control buffer as in (B) for 4 consecutive days. p-p65 (S536) and PUMA in representative tumors were analyzed by Western blotting. (**E**) Paraffin-embedded sections of tumor tissues from mice treated as in (B) were analyzed by TUNEL staining. Left, representative TUNEL staining pictures; Right, TUNEL-positive cells were counted and plotted. (**F**) Tissue sections from (E) were analyzed by active caspase 3 staining. Left, representative staining pictures; Right, active caspase 3-positive cells were counted and plotted. Results of (B), (E) and (F) were expressed as means ± SD of 3 independent experiments. **P < 0.01; *P < 0.05.

Next, we determined whether PUMA-mediated apoptosis is necessary for the antitumor activates of idelalisib in a xenograft model. We established xenograft tumors with parental and *PUMA-*KD HCT116 cells in nude mice. Then tumor-bearing mice were treated with 30 mg/kg idelalisib or the vehicle for 10 days by oral gavage, and tumor volumes were determined every 2 days. This dose of the idelalisib did not significantly lower body weight (Figure 6A), although the mice tended to gain less weight than the control mice. Parental and *PUMA-*KD tumors without treatment were not significantly different in growth (Figure 6B and 6C). The growth of parental tumor was suppressed by 80–90% following idelalisib treatment (Figure 6A and 6B). In contrast, compared to parental, *PUMA-*KD tumors were significantly led to less growth inhibition in response to idelalisib treatment (Figure 6B and 6C), indicating that loss of PUMA abrogated the antitumor effect of idelalisib. PUMA and p65 phosphorylation were increased by idelalisib in xenograft tumors (Figure 6D). TUNEL and cleaved-caspase 3 staining results indicated that significant apoptosis induction in idelalisib-treated parental tumors. However, less positive TUNEL and cleaved-caspase 3 staining were detected in the *PUMA-*KD tumors treated with idelalisib (Figure 6E and 6F). Thus, these data showed that NF-κB/PUMA axis play a key role in the antitumor and apoptotic activities of idelalisib *in vivo*.

## DISCUSSION

Colorectal cancer (CRC) is the most common malignancy with the third largest incidence and mortality among all diagnosed cancers in the worldwide [24]. About 20 to 30% of patients with CRC present metastases when the disease was diagnosis. Moreover, for the remind patients, about 50 to 60% will develop metastases [25]. Right now, CRC accompanied with higher mortality, because of CRC is frequently diagnosed in the advanced stage without reliable biomarkers [26]. Traditional chemotherapy for CRC treatment involves combinations of cytotoxic drugs such as 5-FU, oxaliplatin and irinotecan, and has limited efficacy and substantial side effects due to lack of specificity [27]. Developing of targeted anticancer agents has significantly improved efficacy of chemotherapy against CRC. Idelalisib is a first in class, delta isoform specific, PI3-kinase inhibitor3. Idelalisib targets malignant B-cell proliferation, survival, migration and homing to lymphoid tissues through multiple mechanisms [2, 28, 29]. In the current study, we detected the effect of idelalisib on colon cancer cells. Our results demonstrate for the first time that the therapeutic effect of idelalisib is at least in part mediated by the cell autonomous process of apoptosis induction, progressing from AKT inhibition, GSK3β activation, and p65 nuclear translocation, leading to PUMA induction and onset of mitochondria-dependent apoptosis. p65 not only mediates PUMA induction and apoptotic response to idelalisib, but also is responsible for PUMA-dependent apoptosis induced by aurora kinase inhibitors [17] and regorafenib [16]. In addition to PUMA induction, depletion of Mcl-1 is an early event following idelalisib treatment (Figure 1E), and may also contribute to apoptosis induction [30, 31].

In the present study, idelalisib induces PUMA expression through GSK-3β/NF-κB pathway following AKT inhibition and initiates apoptosis through the intrinsic apoptosis pathway in colon cancer cells. PUMA induction plays a key role in apoptosis in response to varieties chemotherapeutic agents, and is likely to be a useful indicator of chemo sensitivity. Previously reports showed that PUMA induction matches to differential sensitivity to EGFR TKIs, and loss of PUMA induction is associated with insensitive to EGFR TKIs [12, 32]. Furthermore, a recent study demonstrated that response of isolated mitochondria from tumor cells to a peptide containing the Bcl-2 homology 3 (BH3) domains of PUMA correlates with chemotherapy response in patients [33]. The results of the current study suggested that we can check PUMA expression as a biomarker to predict the antitumor effect of idelalisib in colon cancer cells. Although it is hard to get biopsies from colorectal tumors treated with chemotherapy after surgery, it could be possible to detect PUMA induction using non-invasive approaches, such as analysis of circulating tumor cells [34].

In conclusion, our results demonstrated a novel antitumor mechanism of idelalisib through PUMA-mediated apoptosis in a *p53*-independent pathway. Idelalisib-induced PUMA expression change may functions as a biomarker for its clinical trials, and can help important implications for the future development and application.

## MATERIALS AND METHODS

### Cell culture and drug treatment

The human colon cancer cell lines, HCT116, DLD1, HT29, Lim2405, and LoVo were got from American Type Culture Collection (Manassas, VA, USA). All colon cancer cell lines were cultured in DMEM medium supplemented with 10% heat-inactivated newborn calf serum, 100 units/mL penicillin, and 100 μg/mL streptomycin (Invitrogen). NCM356 was got from INCELL (San Antonio, TX), and cultured in M3 media according the supplier's instructions. The anticancer agents and chemicals used include idelalisib, regorafenib (Selleckchem), BAY 11–7082 (Merck), 5-fluoreuracil (5-FU, Sigma) were diluted with DMSO. Constitutively active AKT was got from addgene [35].

### MTS assay

Indicated cell lines were seeded in 96-well plates at a density of 1×10^4^ cells/well. After overnight incubation, various concentrations of idelalisib were added into wells and incubated for additional 72 hr. 3-(4,5-dimethylthiazol-2-yl)-5-(3-carboxymethoxyphenyl)-2-(4-sulfophenyl)-2H-tetrazolium (MTS) assay was performed using the MTS assay kit (Promega) according to the manufacturer's instructions. Luminescence was measured with a Wallac Victor 1420 Multilabel Counter (Perkin Elmer). Each assay was conducted in triplicate and repeated three times.

### Real-time reverse transcriptase (RT) PCR

Total RNA was extracted using the TRIzol RNA Kit (Invitrogen, CA, USA) according to the manufacturer's protocol. One μg of total RNA was used to generate cDNA using SuperScript II reverse transcriptase (Invitrogen). PCR was performed in triplicate using SsoFasr^TM^ Probes Supermix (Bio-Rad) in a final reaction volume of 20 μL with gene-specific primer/probe sets, and a standard thermal cycling procedure (35 cycles) on a Bio-Rad CFX96^TM^ Real-time PCR System. PUMA and β-actin levels were assessed using TaqMan Gene Expression Real-Time PCR assays. Result was expressed as the threshold cycle (Ct). The relative quantification of the target transcripts was determined by the comparative Ct method (ΔΔCt) according to the manufacturer's protocol. The 2^-ΔΔCt^ method was used to analyze the relative changes in gene expression. Control experiments were conducted without reverse transcription to confirm that the total RNA was not contaminated with genomic DNA. β-actin was used as an internal control gene in order to normalize.

### Western blotting

Western blotting was performed as previously described [36, 37], with antibodies for PUMA (Abcam), AKT, phospho-AKT, Bid, cleaved-caspase 3, cleaved-caspase 9, p65, phospho-p65, phospho-FoxO3a, glycogen synthase kinase 3β (GSK3β), phospho-GSK3β, Bak, FoxO3a, cytochrome oxidase subunit IV (Cox IV), p-STAT1, STAT1 (Cell Signaling Technology, Beverly), cytochrome c, lamin A/C, β-actin, Bim (Santa Cruz Biotechnology, Santa Cruz), Mcl-1, and Bcl-XL (BD, San Jose).

### Apoptosis assays

Apoptosis was analyzed by nuclear staining with Hoechst 33258 (Invitrogen) [38]. Annexin V/propidium iodide (PI) staining was performed using annexin-Alexa 488 (Invitrogen) and PI. For analysis of cytochrome c release, cytosolic fractions were isolated by differential centrifugation, and probed by Western Blotting for cytochrome c. For Colony formation assays, the treated cells were plated in 12-well plates at appropriate dilutions and allowing for cell growth for 10 days, followed by crystal violet staining of cell colonies.

### Transfection and siRNA/shRNA knockdown

Cells were transfected with Lipofectamine 2000 (Invitrogen) according to the manufacturer's instructions. Knockdown experiments were performed 24 hours prior to idelalisib treatment using 300 pmole of siRNA. The control scrambled siRNA and siRNA for human p65 (sc-29410), and GSK3β (sc-35527) were from Santa Cruz Biotechnology. For stable transfection a shRNA-expressing plasmid that containing the p53-targeting sequence (CACCATCCACTACAACTACAT) [39], PUMA- targeting sequence (CCTGGAGGGTCATGTACAATCTC TT) [40], or a vector containing a scrambled sequence was transfected into HCT116 cells, followed transfection, cells were plated in 96-well plates in the presence of 5 μg/mL puromycin. The protein expression of puromycin-resistant clones was then analyzed by western blotting.

### Analysis of NF-κB nuclear translocation

HCT116 cells were pre-treated with BAY11-7082 or GSK-3β knockdown, and then subjected to idelalisib treatment for another 6 hours. Nuclear fractionation was used to analyze NF-κB nuclear translocation. Nuclear extracts were isolated from cells using the NE-PER nuclear/cytoplasmic extraction kit (Thermo Fisher) according to the manufacturer's instructions, and analyzed by p65 Western blotting.

### Chromatin immunoprecipitation (ChIP)

ChIP with p65 antibody (Cell Signaling Technology) was performed using the Chromatin Immunoprecipitation Assay Kit (Millipore) according to the manufacturer's instructions. The precipitates were analyzed by PCR using primers 5'-GTCGGTCTGTGTACGCATCG-3' and 5'-CCCGCGTGACGCTACGGCCC-3' to amplify a PUMA promoter fragment containing putative κB sites [16].

### Animal tumor experiments

All animal experiments were performed according to the related ethics regulations of Liaoning University of Traditional Chinese Medicine. HCT116 cells were harvested, and 4 × 10^6^ cells in 0.2 mL of medium were implanted subcutaneously on the back of athymic nude female mice. After tumor growth for 7 days, mice were treated with daily with idelalisib at 30 mg/kg by oral gavage for 10 consecutive days. Tumor growth was monitored by calipers, and tumor volumes were calculated according to the formula ½ × length × width^2^. Mice were euthanized when tumors reached ~1.0 cm^3^ in size. Tumors were dissected and fixed in 10% formalin and embedded in paraffin. TUNEL and active caspase 3 immunostaining was performed on 5 μM paraffin-embedded tumor sections, by using an AlexaFluor 488-conjugated secondary antibody (Invitrogen) for signal detection.

### Statistical analysis

Statistical analyses were carried out using GraphPad Prism IV software. *P* values were calculated by the student's *t-test* and were considered significant if *p* < 0.05. The means ± one standard deviation (s.d.) is displayed in the figures.

## Abbreviations

CRC: colorectal cancer
ChIP: chromatin immunoprecipitation
CLL: chronic lymphocytic leukemia
Cox IV: cytochrome oxidase subunit IV
5-FU: 5-fluoreuracil
E2F1: E2F transcription factor 1
FoxO3a: Forkhead Box O3a
GSK3β: glycogen synthase kinase 3β
IECs: intestinal epithelial cells
LLC: lymphocytic leukemia
NF-κB: nuclear factor κB
PI: propidium iodide
PI3Kδ: phosphatidylinositol 3-kinase delta
PUMA: p53 upregulated modulator of apoptosis
RT-PCR: reverse transcriptase-PCR
STAT1: signal transducer and activator of transcription 1
TUNEL: terminal deoxynucleotidyl transferase mediated dUTP nick end labeling

## References

[1] Hammadi SA, Almarzooqi S, Abdul-Kader HM, Saraswathiamma D, Souid AK (2015). The PI3Kdelta inhibitor idelalisib suppresses liver and lung cellular respiration. International journal of physiology, pathophysiology and pharmacology.

[2] Hewett YG, Uprety D, Shah BK (2016). Idelalisib- a PI3Kdelta targeting agent for B-cell malignancies. Journal of oncology pharmacy practice.

[3] Forcello N, Saraiya N (2014). Idelalisib: The First-in-Class Phosphatidylinositol 3-Kinase Inhibitor for Relapsed CLL, SLL, and Indolent NHL. Journal of the advanced practitioner in oncology.

[4] Furman RR, Sharman JP, Coutre SE, Cheson BD, Pagel JM, Hillmen P, Barrientos JC, Zelenetz AD, Kipps TJ, Flinn I, Ghia P, Eradat H, Ervin T (2014). Idelalisib and rituximab in relapsed chronic lymphocytic leukemia. The New England journal of medicine.

[5] Coutre SE, Barrientos JC, Brown JR, de Vos S, Furman RR, Keating MJ, Li D, O'Brien SM, Pagel JM, Poleski MH, Sharman JP, Yao NS, Zelenetz AD (2015). Management of adverse events associated with idelalisib treatment: expert panel opinion. Leukemia & lymphoma.

[6] Yu J, Wang Z, Kinzler KW, Vogelstein B, Zhang L (2003). PUMA mediates the apoptotic response to p53 in colorectal cancer cells. Proceedings of the National Academy of Sciences of the United States of America.

[7] Yu J, Zhang L. PUMA (2008). a potent killer with or without p53. Oncogene.

[8] Liu Z, Lu H, Shi H, Du Y, Yu J, Gu S, Chen X, Liu KJ, Hu CA (2005). PUMA overexpression induces reactive oxygen species generation and proteasome-mediated stathmin degradation in colorectal cancer cells. Cancer research.

[9] Yu J, Yue W, Wu B, Zhang L (2006). PUMA sensitizes lung cancer cells to chemotherapeutic agents and irradiation. Clinical cancer research.

[10] Dudgeon C, Wang P, Sun X, Peng R, Sun Q, Yu J, Zhang L (2010). PUMA induction by FoxO3a mediates the anticancer activities of the broad-range kinase inhibitor UCN-01. Molecular cancer therapeutics.

[11] Wang P, Qiu W, Dudgeon C, Liu H, Huang C, Zambetti GP, Yu J, Zhang L (2009). PUMA is directly activated by NF-kappaB and contributes to TNF-alpha-induced apoptosis. Cell death and differentiation.

[12] Sun Q, Ming L, Thomas SM, Wang Y, Chen ZG, Ferris RL, Grandis JR, Zhang L, Yu J (2009). PUMA mediates EGFR tyrosine kinase inhibitor-induced apoptosis in head and neck cancer cells. Oncogene.

[13] Ray RM, Bhattacharya S, Johnson LR (2011). Mdm2 inhibition induces apoptosis in p53 deficient human colon cancer cells by activating p73- and E2F1-mediated expression of PUMA and Siva-1. Apoptosis.

[14] Zhang YX, Liu XM, Wang J, Li J, Liu Y, Zhang H, Yu XW, Wei N (2015). Inhibition of AKT/FoxO3a signaling induced PUMA expression in response to p53-independent cytotoxic effects of H1: A derivative of tetrandrine. Cancer biology & therapy.

[15] Ming L, Wang P, Bank A, Yu J, Zhang L (2006). PUMA Dissociates Bax and Bcl-X(L) to induce apoptosis in colon cancer cells. The Journal of biological chemistry.

[16] Chen D, Wei L, Yu J, Zhang L (2014). Regorafenib inhibits colorectal tumor growth through PUMA-mediated apoptosis. Clinical cancer research.

[17] Sun J, Knickelbein K, He K, Chen D, Dudgeon C, Shu Y, Yu J, Zhang L (2014). Aurora kinase inhibition induces PUMA via NF-kappaB to kill colon cancer cells. Molecular cancer therapeutics.

[18] Cross DA, Alessi DR, Cohen P, Andjelkovich M, Hemmings BA (1995). Inhibition of glycogen synthase kinase-3 by insulin mediated by protein kinase B. Nature.

[19] Ding Q, Xia W, Liu JC, Yang JY, Lee DF, Xia J, Bartholomeusz G, Li Y, Pan Y, Li Z, Bargou RC, Qin J, Lai CC (2005). Erk associates with and primes GSK-3beta for its inactivation resulting in upregulation of beta-catenin. Mol Cell.

[20] Inuzuka H, Fukushima H, Shaik S, Liu P, Lau AW, Wei W (2011). Mcl-1 ubiquitination and destruction. Oncotarget.

[21] Thompson PA, Stingo F, Keating MJ, Ferrajoli A, Burger JA, Wierda WG, Kadia TM, O'Brien SM (2016). Outcomes of patients with chronic lymphocytic leukemia treated with first-line idelalisib plus rituximab after cessation of treatment for toxicity. Cancer.

[22] Barr PM, Saylors GB, Spurgeon SE, Cheson BD, Greenwald DR, O'Brien SM, Liem AK, McLntyre RE, Joshi A, Abella-Dominicis E, Hawkins MJ, Reddy A, Di Paolo J (2016). Phase 2 study of idelalisib and entospletinib: pneumonitis limits combination therapy in relapsed refractory CLL and NHL. Blood.

[23] O'Brien SM, Lamanna N, Kipps TJ, Flinn I, Zelenetz AD, Burger JA, Keating M, Mitra S, Holes L, Yu AS, Johnson DM, Miller LL, Kim Y (2015). A phase 2 study of idelalisib plus rituximab in treatment-naive older patients with chronic lymphocytic leukemia. Blood.

[24] Siegel RL, Miller KD, Jemal A (2016). Cancer statistics, 2016. CA Cancer J Clin.

[25] Van Cutsem E, Oliveira J, Group EGW (2009). Advanced colorectal cancer: ESMO clinical recommendations for diagnosis, treatment and follow-up. Annals of oncology.

[26] Newton KF, Newman W, Hill J (2012). Review of biomarkers in colorectal cancer. Colorectal disease.

[27] Goldberg RM (2006). Therapy for metastatic colorectal cancer. The oncologist.

[28] Do B, Mace M, Rexwinkle A (2016). Idelalisib for treatment of B-cell malignancies. American journal of health-system pharmacy.

[29] Cheah CY, Fowler NH, Wang ML (2016). Breakthrough therapies in B-cell non-Hodgkin lymphoma. Annals of oncology.

[30] Tong J, Tan S, Zou F, Yu J, Zhang L (2016). FBW7 mutations mediate resistance of colorectal cancer to targeted therapies by blocking Mcl-1 degradation. Oncogene.

[31] Peng R, Tong JS, Li H, Yue B, Zou F, Yu J, Zhang L (2013). Targeting Bax interaction sites reveals that only homo-oligomerization sites are essential for its activation. Cell death and differentiation.

[32] Misale S, Bozic I, Tong J, Peraza-Penton A, Lallo A, Baldi F, Lin KH, Truini M, Trusolino L, Bertotti A, Di Nicolantonio F, Nowak MA, Zhang L (2015). Vertical suppression of the EGFR pathway prevents onset of resistance in colorectal cancers. Nature communications.

[33] Ni Chonghaile T, Sarosiek KA, Vo TT, Ryan JA, Tammareddi A, Moore Vdel G, Deng J, Anderson KC, Richardson P, Tai YT, Mitsiades CS, Matulonis UA, Drapkin R (2011). Pretreatment mitochondrial priming correlates with clinical response to cytotoxic chemotherapy. Science.

[34] Crowley E, Di Nicolantonio F, Loupakis F, Bardelli A (2013). Liquid biopsy: monitoring cancer-genetics in the blood. Nat Rev Clin Oncol.

[35] Isakova T, Xie H, Messinger S, Cortazar F, Scialla JJ, Guerra G, Contreras G, Roth D, Burke GW, MZ 3rd Molnar, Mucsi I, Wolf M (2013). Inhibitors of mTOR and risks of allograft failure and mortality in kidney transplantation. American journal of transplantation.

[36] Tong JS, Zhang QH, Huang X, Fu XQ, Qi ST, Wang YP, Hou Y, Sheng J, Sun QY (2011). Icaritin causes sustained ERK1/2 activation and induces apoptosis in human endometrial cancer cells. PloS one.

[37] Tong JS, Zhang QH, Wang ZB, Li S, Yang CR, Fu XQ, Hou Y, Wang ZY, Sheng J, Sun QY (2010). ER-alpha36, a novel variant of ER-alpha, mediates estrogen-stimulated proliferation of endometrial carcinoma cells via the PKCdelta/ERK pathway. PloS one.

[38] He K, Chen D, Ruan H, Li X, Tong J, Xu X, Zhang L, Yu J (2016). BRAFV600E-dependent Mcl-1 stabilization leads to everolimus resistance in colon cancer cells. Oncotarget.

[39] Conklin JF, Baker J, Sage J (2012). The RB family is required for the self-renewal and survival of human embryonic stem cells. Nature communications.

[40] Lu H, Hou G, Zhang Y, Dai Y, Zhao H (2014). c-Jun transactivates Puma gene expression to promote osteoarthritis. Molecular medicine reports.

